# Bidirectional chemotherapy allowing surgery and HIPEC for malignant peritoneal mesothelioma

**DOI:** 10.1515/pp-2019-0011

**Published:** 2019-06-21

**Authors:** Barbara Noiret, Clarisse Eveno

**Affiliations:** Department of Digestive and Oncologic Surgery, Claude Huriez University Hospital, Centre Hospitalier Universitaire (CHU) Lille, Université de Lille; INSERM Unité Mixte de Recherche 1172-JPARC Jean-Pierre Aubert Research Center, Team “Mucins, epithelial differentiation, and carcinogenesis”, Lille, France; Department of Digestive and Oncologic Surgery, Claude Huriez University Hospital, Centre Hospitalier Universitaire (CHU) Lille, Université de Lille, Lille, France

**Keywords:** bidirectional chemotherapy, cytoreductive surgery, HIPEC, malignant peritoneal mesothelioma

## Abstract

**Background:**

This case report aims to describe the impact of the bidirectional chemotherapy (BDC) on resecability for initially unresectable malignant peritoneal mesothelioma (MPM).

**Methods:**

We report a case of 55-year-old male with the diagnosis of initially unresecable MPM. The BDC combined intravenous (IV) chemotherapy (Cisplatin–Pemetrexed) and intra peritoneal (IP) chemotherapy (Cisplatin). The response to chemotherapy was assessed by CT – scan and laparoscopy.

**Results:**

Initial evaluation classed the disease as unresecable with PCI at 39. At the reevaluation, CT – scan and laparoscopy showed a macroscopic response, allowing surgery consisting of cytoreductive surgery and hyperthermic intra peritoneal chemotherapy (Doxorubicin and Cisplatin).

**Conclusions:**

BDC (IV and IP) has promising results and allows to undergo surgery for selected patients with borderline or initially unresectable MPM.

We report a case of 55-year-old male with the diagnosis of malignant peritoneal mesothelioma (MPM). Initial evaluation with CT-scan and laparoscopy reveals unresectable peritoneal carcinomatosis with PCI at 39 with thickened omentum (star), small bowel (2 stars) and parietal peritoneum (dash-arrow) deposit, ascitis (plane-arrow) (Figure 1A, B). Bidirectional chemotherapy (BDC) has been performed after three cycles of intravenous (IV) CISPLATIN – PEMETREXED, with intensification combining three cycles of IV PEMETREXED with intraperitoneal (IP) CISPLATIN (Figure 2). At reevaluation, PCI was still at 39 with a macroscopic response (Figure 3A, B). The peritoneal disease was thinner allowing a complete CRS with DOXORUBICIN/CISPLATIN based-HIPEC.

BDC allowed selecting patients with initially unresectable MPM to undergo surgery and increase the overall survival (OS) [1, 2]. New IP delivery with Pressurized IntraPeritoneal Aerosol Chemotherapy (PIPAC) reported promising results in palliative treatment of MMP [3] and is under evaluation to increase OS and secondary resectability of huge MMP (Clinical Trials NCT03875144).

**Figure 1:**
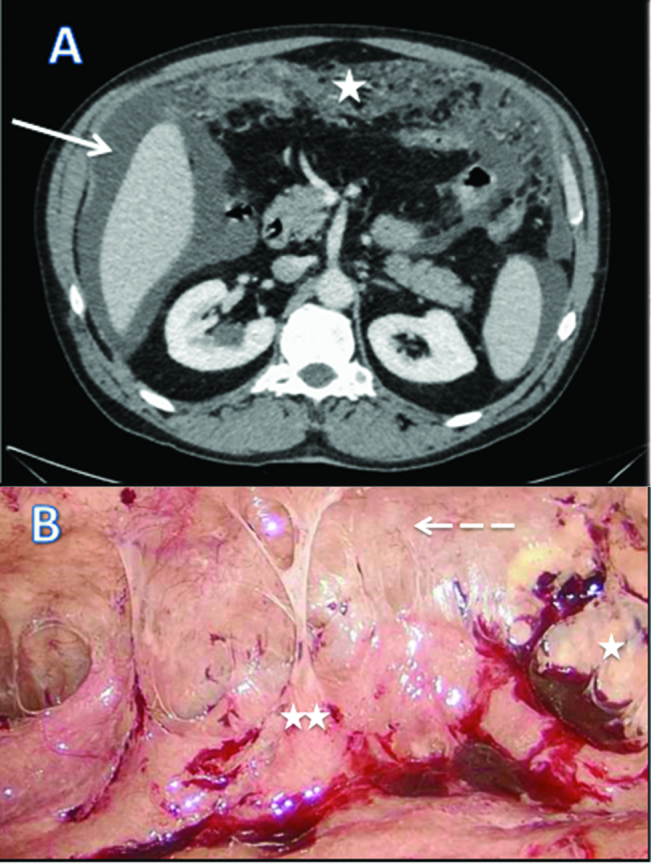
CT scan (A) and laparoscopic evaluation (B) at diagnosis of malignant peritoneal mesothelioma.

**Figure 2:**
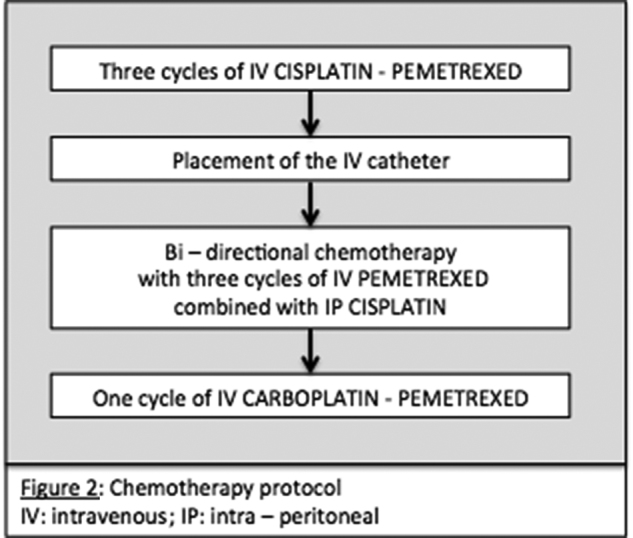
Detailed schedule of chemotherapy.

**Figure 3:**
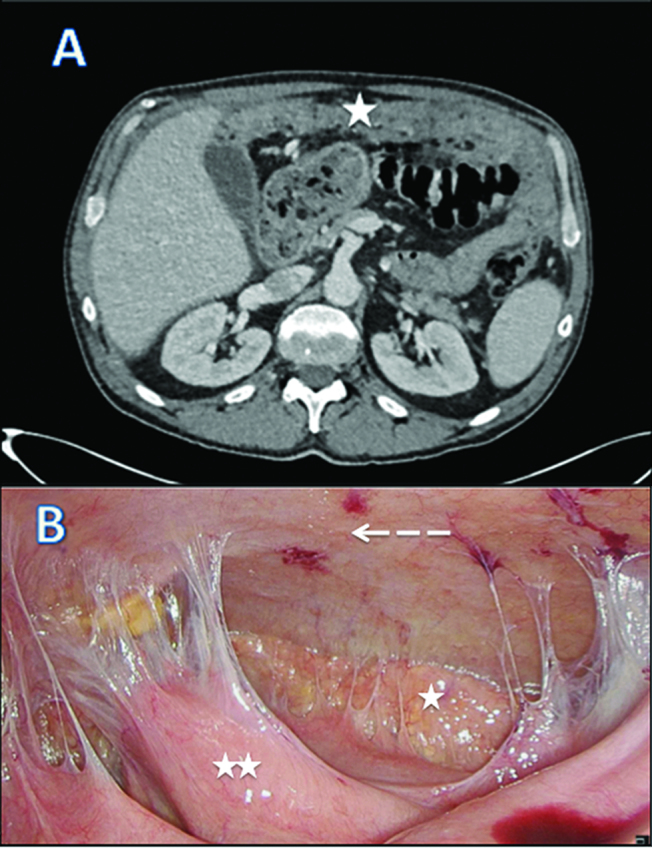
CT scan (A) and laparoscopic evaluation (B) after bi–directional chemotherapy.
